# Predicted Effects of Stopping COVID-19 Lockdown on Italian Hospital Demand

**DOI:** 10.1017/dmp.2020.157

**Published:** 2020-05-18

**Authors:** Jordy Bollon, Matteo Paganini, Consuelo Rubina Nava, Nello De Vita, Rosanna Vaschetto, Luca Ragazzoni, Francesco Della Corte, Francesco Barone-Adesi

**Affiliations:** 1 Department of Translational Medicine, Università del Piemonte Orientale, Novara, Italy; 2 CRIMEDIM – Research Center in Emergency and Disaster Medicine, Università del Piemonte Orientale, Novara, Italy; 3 Department of Economics and Political Science, University della Valle d’Aosta, Aosta, Italy

**Keywords:** COVID-19, intensive care, pandemic, surge capacity

## Abstract

**Objectives:**

Italy has been one of the first countries to implement mitigation measures to curb the coronavirus disease 2019 (COVID-19) pandemic. There is currently a debate on when and how such measures should be loosened. To forecast the demand for hospital intensive care unit (ICU) and non-ICU beds for COVID-19 patients from May to September, we developed 2 models, assuming a gradual easing of restrictions or an intermittent lockdown.

**Methods:**

We used a compartmental model to evaluate 2 scenarios: (A) an intermittent lockdown; (B) a gradual relaxation of the lockdown. Predicted ICU and non-ICU demand was compared with the peak in hospital bed use observed in April 2020.

**Results:**

Under scenario A, while ICU demand will remain below the peak, the number of non-ICU will substantially rise and will exceed it (133%; 95% confidence interval [CI]: 94-171). Under scenario B, a rise in ICU and non-ICU demand will start in July and will progressively increase over the summer 2020, reaching 95% (95% CI: 71-121) and 237% (95% CI: 191-282) of the April peak.

**Conclusions:**

Italian hospital demand is likely to remain high in the next months. If restrictions are reduced, planning for the next several months should consider an increase in health-care resources to maintain surge capacity across the country.

Since December 2019, the coronavirus disease 2019 (COVID-19) has rapidly escalated into a pandemic that is currently threatening national health systems across the world.^1^ To date, with more than 100,000 confirmed cases of COVID-19 and 20,000 deaths,^2^ Italy has also been one of the most affected countries, accounting for the highest death toll in Europe (22%)^3^ and for 15% of the global death toll.^1^ Italy has been also one of the first countries in the world to implement mitigation measures, ie, isolation, quarantine, social distancing, and community containment, in the attempt to curb the COVID-19 pandemic. On March 9, 2020,^4^ a national lockdown was issued, including: (a) strict home confinement for the entire population; (b) closure of all nonessential commercial activities; (c) mobility restrictions between municipalities. As of April 17, approximately 1 month later, the number of infected subjects, intensive care unit (ICU) patients, and non-ICU patients in Italy was 106,962, 2812, and 25,786, respectively. The highest ICU (4068 beds) and non-ICU (29,010 beds) demand was recorded some weeks earlier (April 3 and 4) and then declined, while the number of infected was still on the rise, but approaching a plateau.^2^ Considering that, at the beginning of 2020, there were in Italy approximately 210,000 hospital beds (3.5 beds per 1000 inhabitants) and 5000 ICU beds (0.8 beds per 1000 inhabitants), it is no surprise that the national health system was severely impaired during the early phase of the epidemic. As in other countries, there is currently a debate on when such measures should be loosened, and what can be the best strategy to manage this new phase of the pandemic.^5^ In particular, there are concerns on the effects that an untimely lift of the lockdown could have on health-care systems.

Pandemics are natural disasters characterized by a slow but exponential onset and a prolonged impact.^6^ Even though the gradual increase of cases allows governments to progressively enact mitigation strategies and countermeasures to face an incoming wave, the sustained spread of the disease, along with a possible delayed recovery of patients, can easily lead to health-care capacity saturation and surge capability exhaustion.^7^ In particular, the COVID-19 pandemic has been posing unique challenges to emergency medical services (EMS), emergency departments (ED), and ICUs, in terms of stuff, staff, and structures. Italian ICUs are currently experiencing an unprecedented and sustained demand that is depleting resources on a local, regional, and national levels,^8^ thus possibly hampering further surge in capacity if a new wave of cases should occur. To forecast the demand for hospital ICU and non-ICU beds for COVID-19 patients from May to September, we developed 2 models: (1) assuming gradual easing of restrictions, (2) assuming intermittent lockdown.

## METHODS

### Model Definition

We used a compartmental model to predict hospital demand in Italy associated with the COVID-19 pandemic. A compartmental model divides individuals into groups (or compartments) based on their status and assumes that each individual can only be in a single compartment at a given time. In addition, over the time, an individual can transition between compartments accordingly to transition rates.^9^ The model was implemented using R software, and an overview of it is provided in Supplemental Figure 1 and Table 1. The input to the model is the number of infected individuals in each day of the simulation. Observed numbers of infected individuals were used until April 17 (last available observation). From April 18 onward, the future number of infected individuals under different scenarios (see hereafter) was calculated using the method proposed by Tuite and colleagues.^10^ Based on the number of infected individuals, the model provided the daily number of ICU and non-ICU patients, dead, and recovered. To keep the models as simple as possible, we made a series of assumptions: (i) recovered individuals remained immune from re-infection for the duration of the pandemic; (ii) individuals stopped being infectious once they were admitted to hospital (ie, we did not model transmission within health-care settings).

### Calibration and Validation of the Model

The model was calibrated using the current number of infected, ICU and non-ICU patients, dead, and recovered in Italy from February 24 to March 24, which represents the training data set. These figures were obtained from the website of the Italian Civil Protection.^2^ We used the R function *optim()* to perform a multidimensional optimization of the model, selecting the parameters’ values that minimized the mean square error between fitted and observed number of individuals in each compartment.

In the final model, mean residence times in the different compartments were reasonably consistent with those reported in the scientific literature and those observed in our University Hospital (Maggiore Hospital, Novara). Once the parameters were determined using the training data set, we evaluated the predictive accuracy of the model using the validation data set, comparing the predicted number of subjects in each compartment with the actual figures observed between March 25 and April 17. Supplemental Figure 2 shows that predicted number of subjects in the different compartments were very close to the observed ones, suggesting a satisfactory predictive accuracy of the models.

### Scenario Analysis

We then used our model to forecast the Italian hospital demand until September 1, 2020, under different scenarios:

In Scenario A (“intermittent lockdown”), we first assumed that from April 18 to April 30 the evolution of the pandemic follows the same trend as in the previous weeks, with a steady reduction of new cases of infection over time equivalent to an effective reproductive number (R_t_) of 0.9. Then, we hypothesized that the lockdown is temporarily lifted from May 1 to May 30. Starting from May 31, a new lockdown is enforced until the end of the simulation (September 1), bringing Rt to its original value (0.9). Consequently, we assumed that 2 weeks after the lift, the number of new cases starts to increase again, in a similar manner (R_t_ = 1.3) as it was observed in Italy in the period before the lockdown entered into effect. A lag time of 2 wk was included to take into account the COVID-19 incubation period and the diagnostic delay after the onset of symptoms.^11^

In Scenario B (“gradual relaxation of the lockdown”) we assumed that from May 1 onward, the restrictive measures are progressively reduced over time. Hence, we increased Rt by 0.1 every 30 days, up to the value of 1.3. Assumed Rt values from May 1 to September 1 are reported in Supplemental Figure 3.

We then evaluated changes in ICU and non-ICU demand under the 2 scenarios. ICU and non-ICU needs were compared with the maximum hospital bed use for COVID-19 observed before April 17.

To evaluate the uncertainty of our forecasts, we ran Monte Carlo simulations sampling the values of the parameters from uniform distributions (from -30% to +30% of each parameter’s estimated value). We generated 95% confidence intervals (CIs) by taking the 2.5% and 97.5% percentile estimates from 100 simulations.

### Ethics Committee Approval

The study was based on publicly available aggregate data. No ethics committee approval was necessary.

## RESULTS

### Scenario Analysis

**FIGURE 1:**
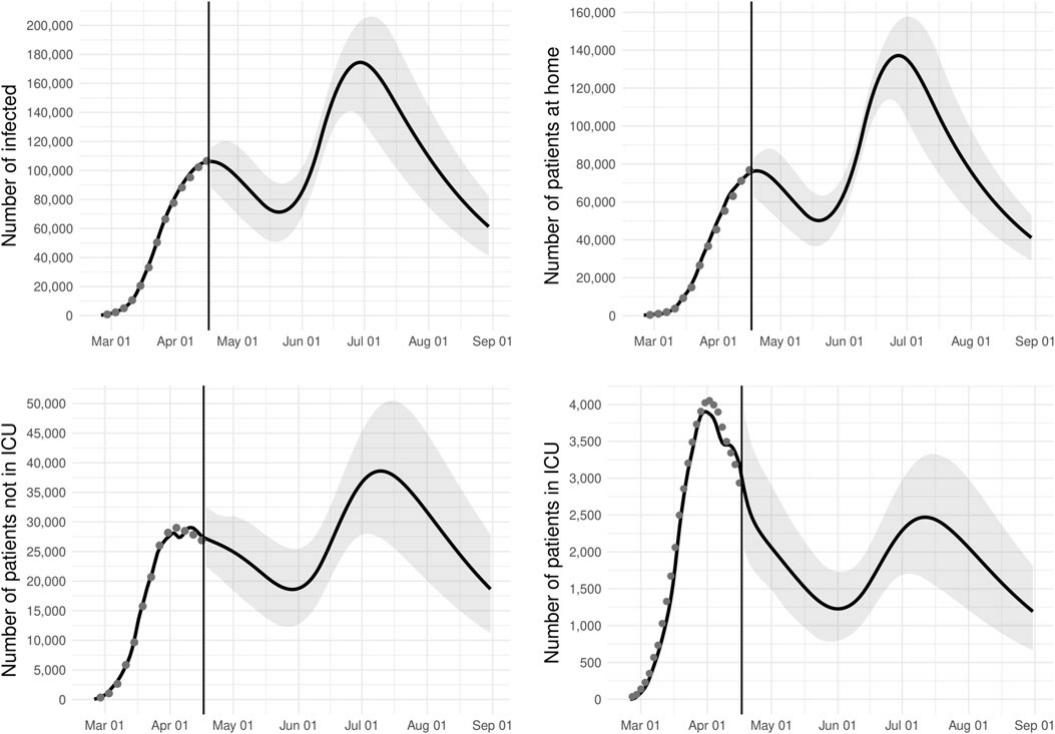
Observed (Circles) and Predicted (Solid Line) Number of Infected, Patients at Home, Non-ICU Hospitalized Patients, and ICU Patients Over Time. Scenario A (intermittent lockdown).

Under scenario A (“intermittent lockdown”), the assumed increase in the number of infected is predicted to translate into a rise in the demand of ICU and non-ICU beds at the beginning of June (Figure 1). The maximum demand of ICU and non-ICU beds will occur in the first weeks of July. While ICU needs will remain below the peak levels observed in April 2020 (61%; 95% CI: 40-81), the number of non-ICU demands will substantially rise and will exceed the maximum demand recorded in the early phase of the pandemic (133%; 95%CI: 94 to 171). Starting from the second part of July, bed demand will start to decrease, but non-ICU needs will still remain high until the end of August.

**FIGURE 2:**
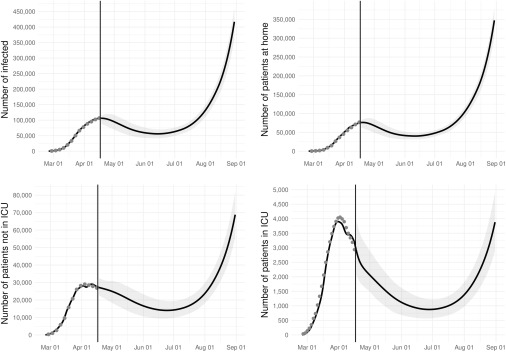
Observed (Circles) and Predicted (Solid Line) Number of Infected, Patients at Home, Non-ICU Hospitalized Patients, and ICU Patients Over Time. Scenario B (gradual relaxation of the lockdown).

Under scenario B (“gradual relaxation of the lockdown”), a rise in the demand of ICU and non-ICU beds will start to be evident in July and will progressively increase over the summer (Figure 2). At the end of August, ICU and non-ICU demand will be 95% (95% CI: 71-121) and 237% (95% CI: 191-282) of the April peak. Differently from the previous scenario, no reduction in both ICU and non-ICU demand is predicted during the time frame covered by the simulation.

## DISCUSSION

In this study, we showed that an early reduction of the community containment measures currently in place in Italy could shortly translate into a substantial increase of ICU and non-ICU demand. In particular, a gradual lift of the lockdown (which is currently under discussion by the Italian government) is expected to double non-ICU admissions and to bring the number of ICU needs to the same levels observed in the early phase of the pandemic.

Our results indicate that the Italian health system should focus efforts to prepare for the next phase of the COVID-19 pandemic. Within the hospital system, critical care services are fundamental during epidemics of infectious respiratory diseases,^6^ as repeatedly demonstrated during H1N1, severe acute respiratory syndrome (SARS), or Middle East respiratory syndrome (MERS) outbreaks. During the early stage of an epidemic, a phased response can be useful to gradually increase treatment capacity while preserving critical care resilience and preventing the collapse of the system. Similarly, a phased deactivation of resources is of paramount importance during the recovery stage of epidemics, because the risk of new outbreaks is consistent^12^ and the scarce, remaining assets should be properly managed.

Our analysis also shows that non-ICU demand could become a rate-determining step for the health systems, because of the length of stay in the wards and the step-down of patients from the ICU. The former could be partly due to the commonly used discharge criteria for COVID-19 cases. The European Centre for Disease Prevention and Control (ECDC) currently recommends clinical resolution and 2 negative tests for discharge.^13^ However, ECDC recognizes that, in the context of sustained widespread transmission (as it is currently the case in Italy), hospital discharge should be also based on other factors, such as the existing capacity of the health-care system, laboratory diagnostic resources, and the current epidemiological situation.^13^ In particular, the earlier discharge from hospital of mild cases, if clinically appropriate, may be considered, provided that they are placed into home care or another type of community care and periodically evaluated.^13^ Telemedicine could be also useful to foster early discharge of patients, thus helping to keep non-ICU demand under control.

Our model is based on several assumptions and has limitations. In particular, we assumed that the trend of non-ICU and ICU admission rates in the next months will remain similar to what we observed so far. Thus, for example, an improvement in the community-based management of COVID-19 patients would translate into a reduction of hospital demand. Moreover, we did not take into account the effect that the gradual depletion of susceptible persons from the population would have on our estimates. However, considering that the prevalence of infected persons is still low in Italy (<10%), this simplification should not have substantially affected our results.^14^

In conclusion, our results suggest that Italian hospital demand is likely to remain high in the next months if restrictions are reduced, which seems likely to occur. Given the cuts recently suffered by the Italian National Health System,^15^ planning for the next few months should consider an increase in health-care resources to maintain surge capacity across the country. The available assets should be deployed to the most struggling parts of the country with a certain grade of flexibility over time, taking also into account the immunity status of the population.

## Supplementary material

For supplementary material accompanying this paper visit http://dx.doi.org/10.1017/dmp.2020.157. click here to view supplementary material
