# Supplementary material for: Predicted Effects of Stopping COVID-19 Lockdown on Italian Hospital Demand
Source: Disaster Med Public Health Prep. 2020 May 18:1–5. doi: 10.1017/dmp.2020.157 (PMC7276503; doi:10.1017/dmp.2020.157)
Supplement: Supplementary file 1 [file S1935789320001573sup.zip › S1935789320001573sup002.docx]

**Suppl. Table 1.** Parameters of the model.

| **Parameter** | **Value** |
| --- | --- |
| A (hospitalization rate) | Declining over time from 41% to 2.3% per day during the first 54 days (observed time frame) |
| B (rate of ICU admission) | Declining over time from 8% to 1.5% per day during the first 54 days (observed time frame) |
| C (discharge rate from ICU) | 0% during the first 4 days of staying in ICU, then 22% per day |
| D (mortality rate in non-ICU wards) | 1.1% per day |
| E (mortality rate in ICU) | 13% per day |
| F (hospital discharge rate) | 0% during the first 6 days of hospital stay, then 5.3% per day |
| G (recovery rate for not hospitalized patients) | Subjects recover 29 days after the diagnosis. |
