# Supplementary material for: Predicted Effects of Stopping COVID-19 Lockdown on Italian Hospital Demand
Source: Disaster Med Public Health Prep. 2020 May 18:1–5. doi: 10.1017/dmp.2020.157 (PMC7276503; doi:10.1017/dmp.2020.157)
Supplement: Supplementary file 1 [file S1935789320001573sup.zip › S1935789320001573sup001.docx]

**Suppl. Figure 2.** Validation of the model. Predicted and observed values in the different compartments.
