# Supplementary figures and images for: Predicted Effects of Stopping COVID-19 Lockdown on Italian Hospital Demand
Source: Disaster Med Public Health Prep. 2020 May 18:1–5. doi: 10.1017/dmp.2020.157 (PMC7276503; doi:10.1017/dmp.2020.157)

**Suppl. Figure 3.** Changes of the effective reproductive number (Rt) under the assumed scenarios.


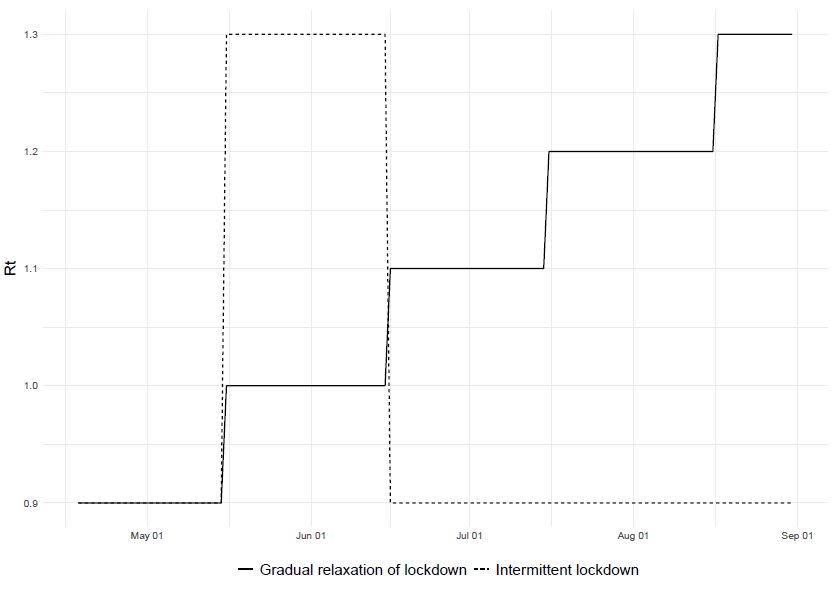

Supplement: Supplementary file 1 [file S1935789320001573sup.zip › S1935789320001573sup003.docx]
